# Quantification of the demands of cricket bowling and the relationship to injury risk: a systematic review

**DOI:** 10.1186/s13102-021-00335-8

**Published:** 2021-09-10

**Authors:** Matthew Constable, Daniel Wundersitz, Rodrigo Bini, Michael Kingsley

**Affiliations:** 1 Holsworth Research Initiative, La Trobe Rural Health School, La Trobe University, Bendigo, VIC Australia; 2 Department of Exercise Science, Faculty of Science, The University of Auckland, Auckland, New Zealand

## Abstract

**Background:**

Bowling in cricket is a complex sporting movement which, despite being well characterised, still produces a significant number of injuries each year. Fast bowlers are more likely to be injured than any other playing role. Frequency, duration, intensity and volume of bowling, which have been generalised as measurements of workload, are thought to be risk factors for injuries. Injury rates of fast bowlers have not reduced in recent years despite the implementation of various workload monitoring practices.

**Objective:**

To identify the variables used to quantify frequency, intensity, time and volume of bowling; and evaluate relationships between these variables and injury risk.

**Methods:**

Six online databases were systematically searched for studies on fast bowling that included terms related to workload. Population characteristics, variables relating to demand and their relationship to standardised definitions of physical activity were extracted from all included studies.

**Results:**

Bowling workload is typically quantified through measures of frequency, duration, or indirect intensity, with few studies reporting on bowling volume.

**Conclusions:**

When reported on, volume was often described using imprecise or insufficient measures of intensity. There is a need to develop more appropriate measures of intensity during bowling and improve the quality of evidence to inform on bowling programme management practices.

## Key points

- The incidence and prevalence of injuries in cricket fast bowlers remain high despite the introduction of bowling guidelines that aim to reduce the risk of injury.
- The use of the term workload in bowling is imprecise in reflecting forces or how bowling volumes are implemented or monitored.
- A requirement exists to improve methods for calculating bowling intensity and volumes by including a measure of force to quantify acute and chronic demands of bowling to provide greater insight into injury risk and management.

## Background

Cricket is a popular bat and ball sport played worldwide that is highly lucrative at the elite level [1]. There are three common formats of the game that vary in duration from 3 h in T20 cricket of 20 overs per side, to multi-day cricket played over 3–5 days of 90–100 overs per day. The demands vary considerably across the three formats with more intense physical activity per unit of time experienced during limited overs matches and higher levels of total demand experienced during multi-day matches [2]. Across all formats, fast bowlers accumulate the largest physiological demands [2]. This is due to the complex nature of the bowling action that imposes high horizontal and vertical ground reaction forces (GRF) on the body, as well as shoulder counter-rotation, rapid lateral flexion, and rotation of the lumbar spine [3]. These factors result in an elevated risk of injury in bowlers [4], with the prevalence of injury in fast bowlers greater than 20% and much higher compared to the next most injured (< 10%) player role [4–6]. Lower back injuries are the most common injury with the most serious being stress fractures of the lumbar spine [4]. These injuries require significant rehabilitation, impact on player mental health, financially burden players and result in extended absences from the game [7].

Previous research has identified two key factors associated with lumbar spine injuries [8]. One relates to the bowling action and the extreme movements involved [9, 10], such as shoulder counter-rotation, lateral flexion, or rotation of the spine [11]. The second relates to the demands of bowling, typically expressed generically by the term ‘workload’ [12–27]. However, in sports science and medicine, the demands of physical activity and exercise are quantified using the frequency, intensity, time, type, volume and progression (FITT-VP) framework advocated by the American College of Sports Medicine (ACSM) [28]. While the term workload is used frequently in both theoretical and practical contexts as a generalised definition of effort, its use to describe the demands of bowling is imprecise for reasons highlighted previously by Knuttgen [29]. For example, the force (N) imparted on a cricket ball when bowled multiplied by the distance over which the force is applied yields ‘work’ (measured in joules; J). ‘Load’, however, only exists if there is some form of resistance (also expressed as N) against the work being performed. In this example the ‘load’ on the cricket ball has two possible components which are wind resistance, and friction introduced by the ball bouncing on the pitch, in transit to the batsman. Neither of these relate to the physical demands experienced by the bowler. The use of workload considered without aspects of force and resistance experienced by the bowler are therefore not useful in explaining demand effects on the body or injury risk. The simple counting of balls, for example, is less useful from a demand perspective due to the variations possible between bowlers with respect to bowling technique, running speed, and anthropometrical characteristics such as height and mass. Another problem with the term workload is the variability with which it is used in the literature. For example, studies report bowling frequency [17, 19–21, 24, 26, 30–35], duration [25, 36, 37] or intensity [12, 14, 16, 23, 31, 35, 36, 38–50] when describing bowling workloads. None of these can measure workload in isolation and the term workload, when used as is, is not sufficiently precise for describing bowling programmes or in improving injury monitoring practices. We believe, a more appropriate generic term for what is being measured would be *demand*. When frequency, duration and intensity are combined, an accumulated bowling demand can be quantified over a session, week, season or career, which is bowling volume [2, 12, 13, 15, 16, 18, 22, 23, 27, 41, 42, 47–49, 51–59]. Variability in the methods used to quantify the demands of bowling have led to the development of bowling monitoring practices that have not improved injury rates [4]. Frequency and time quantify how often and for how long an activity is completed, respectively. Type describes the exercise or activity being performed and progression describes how activity is progressed to continually achieve gains in fitness, form or function. Most importantly within the context of injury management, intensity quantifies effort by describing how hard an activity is being performed [60] and intuitively provides greater meaning within this context. Volume, as the product of frequency, duration and intensity [28], is important because frequency and time on their own provide no meaningful estimation of effort and are therefore, inadequate in managing injury risk.

The use of mainly frequency and time-based measures to manage bowling programmes is common, with bowling guidelines established from grassroot to elite levels of cricket [61]. The strictest guidelines are applied to underage groups where research has demonstrated that players are at greatest risk of developing lumbar spine injuries mainly due to physiological immaturity [13, 25, 30, 32, 62]. However, the incidence and prevalence of lumbar spine injuries in bowlers, across all age groups, has not significantly improved since the implementation of these guidelines [4].

The primary aim of this review was to identify the variables used to quantify frequency, intensity, time and volume of bowling. The secondary aim was to investigate relationships between these variables and risk of injury.

## Methods

### Protocol

This systematic review was performed and reported in accordance to the guidelines described by The PRISMA 2020 statement [63].

### Inclusion criteria

Studies were included if they tested human participants, published in English, original research or peer-reviewed, related to the full bowling action, used some variable measuring frequency, time, intensity or volume, and used validated measurement methods.

### Search strategy

Keyword search terms were cricket, which was combined using the Boolean AND operator with bowl*, move*, force*, load*, work*. Filters were used in some databases to reduce the number of non-relevant studies (e.g., non-human or non-English studies). The literature search included all documents from inception to 28th April 2021. Six online databases were searched initially (Medline, Embase, Scopus, SPORTDiscus, CINAHL and AUSPORT), followed by a supplementary search that included Google Scholar. The reference lists of all included articles were also examined to determine if all relevant articles had been found.

### Study selection

All references were exported to Endnote (X9, Clarivate, USA) and duplicates removed. Two reviewers (MC, DW) screened titles and abstracts as per the inclusion criteria and retrieved full text for further analysis. Disagreements were resolved by a third reviewer (MK).

### Data extraction

Data extracted included demographic information, study methodology, study focus, and variables commensurate with the ACSM definitions of frequency, time, intensity or volume [64]. Extracted variables were not always able to be separated between bowler classifications due to the way in which results were reported, however, as our specific focus was on the methods used to quantify the demands of bowling, data were extracted for both bowler categories.

### Risk of bias and quality of evidence assessment

Neither risk of bias or quality of evidence assessment were conducted as the purpose of this review was not to summarize the findings of included studies or how bias may be introduced into the results of these studies. The purpose was to primarily summarize the methods used to quantify frequency, intensity, time and volume of bowling in cricket-based studies rather than critique the reported results.

## Results

### Study selection

**Fig. 1:**
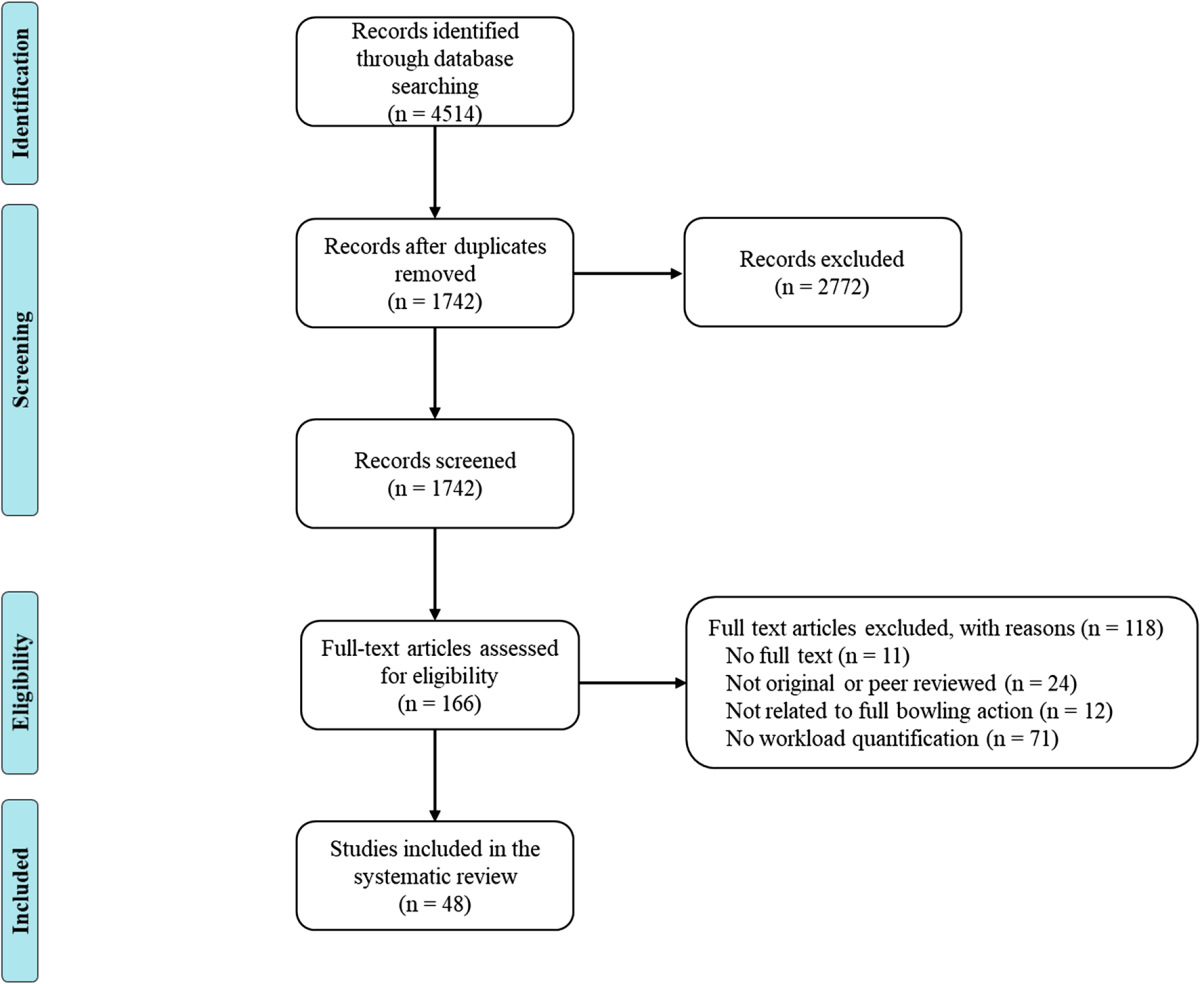
Preferred Reporting Items for Systematic reviews and Meta Analyses (PRISMA) flow chart of included studies

The initial search identified 4514 articles from online databases and Google Scholar. Full text screening was performed on 166 studies with 48 of these included in the systematic review (Fig. 1).

### Study characteristics

Of the 48 studies included for review, twenty-two [2, 31, 33–35, 38–40, 42–46, 48–50, 52–55, 57, 58] did not report any workload or intensity variable as primary outcomes (Table 1). Rather, these studies used variables as a means of ensuring consistency of performance (e.g., ball release velocity) or as a construct of the protocol (e.g., balls bowled). Seventeen studies were prospective cohort studies, reflecting on data recorded across periods of time varying from a single match to 10 years and described demands of match play [56], injury or injury risk [18, 23, 30, 32, 36], bowling frequency and injury [17, 20, 21, 24–26, 37], bowling volume and injury [15], talent identification [33] or bowling ‘workload’ [12, 27]. Fourteen studies were observational cohort studies describing bowling kinematics [2, 31, 34, 55], physiology or physiological profiles of players [16, 22, 38, 39, 46, 48], bowling characteristics using microtechnology [41, 52, 58], or some combination of these [47]. Five studies used a longitudinal observational study design to describe movement patterns [53, 54], physiological demands of cricket [45, 65], or bowling volume management [13]. Studies with other study designs examined performance variability [42], physiology and performance [43, 44, 50], performance [14], quantifying bowling volume with microtechnology [51, 59], validation of microtechnology [40], coaching practices [19], movement patterns [57], kinematics of bowling [49], and exercise-based injury prevention [35].

**Table 1:** Characteristics of studies and variables used for workload estimation

Study	Design type	N	Participant characteristics (bowler type; level; age; gender; country of origin)	Study focus	FITT-VP classification of extracted variables
Alway et al. [17]	Prospective cohort	368	Fast bowlers; elite; 25 ± 6 yr; male; UK	Injury risk	*Frequency—*Balls bowled
Bayne et al. [30]	Prospective cohort	25	Fast bowlers; mixed; Inj: 16 ± 1 yr, Non-inj: 16 ± 1 yr; male; Australia	Injury	*Frequency—*Balls bowled
Bliss et al. [65]	Longitudinal observational	13	Seam bowlers; elite; 28 ± 4.2 yr; male; UK	Physiological match demands	*Volume* – GPS measured distance and accelerations
Burnett, Elliott & Marshall [31]	Observational cohort	9	Fast bowlers; emerging elite; 18 ± 1 yr; male; Australia	Fatigue effects on bowling technique	*Frequency—*Balls bowled *Intensity—*Blood lactate; heart rate; BRV
Cooke et al. [16]	Observational cohort	22	Bowler type NS; elite; 24 ± 9 yr; male; UK	Differences in fatigue	*Frequency—*Balls bowled *Intensity—*RPE *Volume—*Playerload™
Dennis et al. [26]	Prospective cohort	90	Fast bowlers; elite; 18–38 yr; male; Australia	Injury risk	*Frequency—*Balls bowled
Dennis et al. [25]	Prospective cohort	44	Fast bowlers; sub-elite; 15 ± 1 yr; male; Australia	Injury risk	*Frequency—*Bowling logbook
Dennis et al. [36]	Prospective cohort	91	Fast bowlers; emerging elite; 12–33 yr; male; Australia	Injury risk identification	*Frequency—*Bowling logbook; balls bowled *Intensity—*BRV
Duffield et al. [38]	Observational cohort	6	Med-fast bowlers; elite; 23 ± 3 yr; male; Australia	Physiological response & performance	*Intensity—*BRV; RPE
Feros et al. [50]	RCT	12	Fast bowlers; sub-elite; 23.7 ± 7.5 yr; male; Australia	Physiology and performance	*Intensity* – BRV; RPE
Forrest et al. [35]	Cluster-RCT	65	Fast bowlers; sub-elite; 15.6 ± 6 yr; male; Australia	Injury risk factors	*Frequency* – Bowling logbook *Intensity* – RPE
Forrest et al. [49]	Cluster-RCT	64	Fast bowlers; sub-elite; 15.6 ± 6 yr; male; Australia	Kinematics and injury risk	*Intensity*—RPE *Volume* – GPS measured distance
Gabbett et al. [15]	Prospective cohort	28	Fast bowlers; elite; 26 ± 5 yr; male; Australia	Injury risk	*Volume—*ACWR
Garcia-Byrne et al. 2020 [59]	Retrospective cohort	34	Fast bowlers; elite and emerging elite; elite 26 ± 4 yr, emerging elite 16 ± 1 yr; female, Australia	Load measuring using technology	*Volume* – GPS measured distance; Playerload™
Gregory et al. [32]	Prospective cohort	112	70 fast bowlers, 42 spin bowlers; emerging elite; 12–21 yr; male; UK	Injury comparison	*Frequency—*Balls bowled
Gregory et al. [37]	Prospective cohort	70	Fast bowlers; emerging elite; 15 ± 2 yr; male; UK	Injury risk	*Frequency –* Bowling logbook; Balls bowled
Greig & Child [14]	Repeated measures, field based	12	Fast bowlers; emerging elite; 19 ± 1 yr; male; UK	Performance & loading	*Intensity—*BRV *Volume—*Playerload™
Greig & Nagy [51]	Repeated measures, field based	10	Fast bowlers; emerging elite; 18 ± 1 yr; male; UK	Efficacy of technology in load quantification	*Volume* Playerload™
Hulin et al. [23]	Prospective cohort	28	Fast bowlers; elite; 26 ± 5 yr; male; Australia	Injury risk	*Frequency—*Balls bowled *Intensity—*RPE *Volume—*ACWR
Johnstone et al. [39]	Observational cohort	7	Fast-medium bowlers; elite; 25 ± 5 yr; male; UK	Physiological profiles of bowlers	*Intensity—*Heart rate
Jowitt et al. [58]	Cross-sectional	35	Fast bowlers; elite; 18–35 yr; 30 male, 5 female; UK	Detection of bowling events using technology	*Volume* – GPS measured distance
McGrath et al. [40]	Cross-sectional	17	Bowler type NS; sub-elite; Age NS; male; New Zealand	Detection of bowling events using technology	*Intensity—*BRV
McNamara et al. [22]	Observational cohort	26	9 fast bowlers, 17 non-fast bowlers; emerging elite; 18 ± 1 yr; male; Australia	Fatigue response	*Volume—*Playerload™
McNamara et al. [52]	Observational cohort	12	Fast bowlers; mixed; 24 ± 4 yr; male; Australia	Detection of bowling events using technology	*Volume—*Playerload™
McNamara et al. [42]	Repeated measures observational	7	Fast bowlers; elite; 22 ± 3 yr; male; Australia	Performance variability	*Intensity—*BRV *Volume—*Playerload™
McNamara et al. [41]	Observational cohort	12	Fast bowlers; elite; 20 ± 2 yr; male; Australia	Load measuring using technology	*Intensity—*BRV *Volume—*Playerload™
Minett et al. [44]	Randomised, repeated measures, cross-over	10	Med-fast bowlers; mixed; 23 ± 8 yr; male; Australia	Physiology and performance	*Intensity—*BRV
Minett et al. [43]	Randomised, repeated measures, cross-over	8	Med-fast bowlers; mixed; 23 ± 5 yr; male; Australia	Physiology and performance	*Intensity—*BRV
Orchard et al. [24]	Prospective cohort	129	Fast bowlers; elite; Age NS; male; Australia	Injury risk	*Frequency—*Balls bowled
Orchard et al. [20]	Prospective cohort	235	Fast bowlers; elite; Age NS; male; Australia	Injury risk	*Frequency—*Balls bowled
Orchard et al. [21]	Prospective cohort	235	Fast bowlers; elite; Age NS; male; Australia	Injury risk	*Frequency—*Balls bowled
Patel et al. [33]	Prospective cohort	438	Bowler type NS; emerging elite; Age NS; male; New Zealand	Talent identification	*Frequency—*Balls bowled
Petersen et al. [55]	Observational cohort	1	Bowler type NS; elite; Age NS; male; Australia	Variability in movement patterns	*Volume—*GPS measured distance
Petersen et al. [53]	Longitudinal observational	18	Bowler type NS; elite; Age NS; male; Australia	Quantification of positional movement patterns	*Volume—*GPS measured distance
Petersen et al. [2]	Observational cohort	42	Bowler type NS; emerging elite; 22 ± 3 yr; male; Australia	Variability in movement patterns	*Volume—*GPS measured distance
Petersen et al. [54]	Longitudinal observational	54	Bowler type NS; elite; International: 30 ± 4 yr, State*:* 27 ± 3 yr; male; Australia	Comparison of movement patterns between 1-day and test matches	*Volume—*GPS measured distance
Petersen et al. [45]	Longitudinal observational	42	Bowler type NS; emerging elite; 22 ± 3 yr; male; Australia	Training and game demands	*Intensity—*Blood lactate; heart rate *Volume—*GPS measured distance
Pote & Christie [13]	Longitudinal observational	12	Bowler type NS; emerging elite; 16–19 yr; male; South Africa	Workload management	*Frequency—*Balls bowled *Intensity—*RPE *Volume—*ACWR
Rowlands et al. [34]	Observational cohort	NS	Bowler type NS; not specified; not specified; male; Australia	Bowling action analysis	*Frequency –* Balls bowled (not reported in results)
Sholto-Douglas et al. [57]	Retrospective cohort	7	Fast bowlers; elite; Age NS; male; Australia	Movement patterns in T20 games	*Volume* – GPS measured distance
Soomro et al. [19]	Survey	548	All players; mixed; Age NS; male; Australia	Coaching workload management practices	*Frequency—*Balls bowled
Stretch & Lambert 1999 [46]	Observational cohort	21	Fast bowlers; emerging elite; Junior: 12–13 yr, Senior: 18–22 yr; male; Australia	Fatigue response	*Intensity—*Heart rate
Tallent et al. [12]	Prospective cohort	8	Bowler type NS; elite; 22 ± 3 yr; male; UK	Quantification of workload and cognitive function	*Intensity –* VAS *Volume—*GPS measured distance
Tysoe et al. [27]	Prospective cohort	45	Fast bowlers; elite; 27 ± 5 yr; male; UK	Workload management	*Volume*—ACWR
Vickery et al. [48]	Observational cohort	11	Fast/spin bowlers; sub-elite; 22 ± 4 yr; male; Australia	Physiological responses and movement demands	*Intensity—*Blood lactate; heart rate; RPE *Volume—*GPS measured distance
Vickery et al. [47]	Observational cohort	18	Fast-med bowlers; elite; 21 ± 4 yr; male; Australia	Association of internal and external measures of load	*Intensity—*Heart rate; RPE *Volume—*Playerload™
Vickery et al. [56]	Prospective cohort	42	Fast/spin bowlers; emerging elite; 23 ± 4 yr; male; Australia	Comparison of training and match play physical demands	*Intensity—*Heart rate; RPE *Volume—*GPS measured distance
Warren et al. [18]	Prospective cohort	23	Fast bowlers; emerging elite; 17 ± 1 yr; male; UK	Injury risk	*Frequency—*Balls bowled *Volume—*ACWR

Age reported as mean ± SD or as a range. Note: ACWR—acute:chronic workload ratio; BRV—ball release velocity; FITT-VP—Frequency, intensity, time, type, volume, progression; GPS—global positioning system; NS—Not specified; RPE—rating of perceived exertion; UK—United Kingdom; VAS—visual analogue scale

Age groups of participants were not reported in some studies [13, 19–21, 24, 33, 34, 53, 55, 57] but adult cohorts were most common [2, 12, 14–17, 23, 26, 27, 38, 39, 41–45, 47, 48, 50–52, 54, 56, 58, 65], with underage [13, 18, 22, 25, 30, 31, 35, 37, 49] and mixed age cohorts [32, 36, 46, 59] also used. Cohorts were drawn from international and first-class squads [12, 15–17, 20, 21, 23, 24, 26, 27, 38, 39, 41, 42, 47, 53–55, 57–59, 65], members of emerging talent squads and programmes [2, 13, 14, 18, 22, 31–33, 36, 37, 45, 46, 51, 56, 59], mixed squads [19, 30, 43, 44, 52, 59], or sub-elite squads only [25, 34, 35, 40, 48–50]. Participants in the included studies were mostly fast or fast-medium bowlers; spin bowlers were included in seven studies [16, 17, 32, 48, 51, 54, 55].

### FITT-VP variables

Twenty-eight studies included the use of only a single bowling demand variable [2, 15, 17, 19–22, 24–27, 30, 32–34, 39, 40, 43, 44, 46, 51–55, 57, 58, 65], eleven studies used two [12, 14, 18, 35, 37, 38, 41, 49, 50, 52, 59] or three variables [13, 16, 23, 36, 45, 47, 56], and two studies used four variables [31, 47]. The most common combination of variables used were heart rate (HR) and blood lactate (BL) [31, 45, 48], balls bowled and rating of perceived exertion (RPE) [13, 16, 23], balls bowled and acute: chronic workload ratio (ACWR) [13, 18, 23], HR and global positioning system-derived (GPS) variables [45, 48, 56], and HR and RPE [47, 48, 56]. No other combination of variables was used more than twice. Studies not related to injury did not report on any variable of intensity as an outcome but rather used these variables to ensure standardisation of participant effort across trials. Most variables extracted from the included studies were classified as objective and non-physiologically based (Table 2). Objective and physiological variables of intensity used were limited to HR and BL, and these were typically used in studies examining the effects of fatigue on bowling performance and action [19, 31], or to ensure consistency of participant effort [39, 45, 47, 48, 56]. The main subjective measure of intensity reported was RPE [13, 16, 23, 35, 38, 47–50, 56] with a visual analogue scale (VAS) [12] only used in one study to measure changes in cognition after a spell of bowling.

**Table 2:** Summary of extracted bowling variables

Variable	Studies report (n)	Variable FITT-VP classification
*Objective, physiological*		
Heart rate [31, 39, 45–48, 56]	7	Intensity
Blood lactate [31, 45, 48]	3	Intensity
*Objective, non-physiological*		
Balls bowled [13, 16–21, 23, 24, 26, 30–34, 36, 37]	17	Frequency
GPS variables (speed/distance) [2, 12, 45, 48, 49, 53–59, 65]	13	Volume
BRV [14, 31, 36, 38, 40–44, 50]	10	Intensity
Playerload™ [14, 16, 22, 41, 42, 47, 51, 52, 59]	9	Volume
ACWR [13, 15, 18, 23, 27]	5	Volume
Bowling logbook [25, 35–37]	4	Frequency & time
*Subjective, physiological*		
RPE [13, 16, 23, 35, 38, 47–49, 56]	9	Intensity
*Subjective, non-physiological*		
VAS [12]	1	Intensity

ACWR—acute:chronic workload ratio; BRV—ball release velocity; FITT-VP—Frequency, intensity, time, type, volume, progression; GPS—global positioning system; RPE—rating of perceived exertion; VAS—visual analogue scale

### FITT-VP variables and injury

Fourteen studies investigated FITT-VP variables and their relationship to injury risk. Five studies evaluated the relationship between ACWR and injury risk [13, 15, 18, 23, 37]. A further three studies examined injury rate and risk where variations in bowling existed either between-bowlers [25, 26] or within-bowlers [17]. Of these studies, two suggested benefits in reduction of injury risk when using the dual-threshold approach [25, 26]; the remaining study found that acute spikes in bowling were likely to increase the risk of injury [17]. Three additional studies looked at historical injury statistics, and the number of balls bowled by each bowler, obtained from official records of matches played in the Australian first-class competition between 1998/1999 and 2012/2013 inclusive [20, 21, 24]. These studies found that tendon injuries were more likely to occur with sudden increases in the quantity of balls bowled [21]; that although exceeding 100 overs during a period of less than 17 days increased injury risk, less than 100 overs in a 12–26 day period did not significantly increase injury risk [20]; and high acute number of overs may lead to a delayed risk of injury of 2 to 4 weeks [24]. Another single study used field-based tests and bowling technique analyses to identify biomechanical components of the bowling action that might predict injury [36]. Finally, two closely related studies investigated the effectiveness of an exercise-based injury prevention program [35], and the modification of bowling kinematics through the same exercise-based injury prevention program [49], in reducing injury risk.

## Discussion

It is well-established that cricket fast bowlers carry the largest physical demand in cricket and are subject to a greater injury risk than other players [4]. Considering this, we systematically searched the literature related to cricket bowling and synthesised information related to the variables of frequency, time, intensity and volume used to monitor bowling.

Within the literature, bowling frequency [13, 16–21, 23–26, 30–37] and intensity [12–14, 16, 23, 31, 35, 36, 38–46, 48–50, 56] were the most commonly reported variables. Frequency was typically measured as the number of balls bowled in a training session/ match, or as part of a logbook which captured bowling over one or more seasons. Logbooks were used to record the number of balls bowled and sometimes included a duration (time) of how long a bowler bowled for in each session or match. In isolation, variables of frequency and time, without a measure of intensity, can only give an overview of bowling demand and cannot be used to calculate volume. This is important because without an understanding of the intensity level a bowler is bowling at the link to injury risk is likely to be tenuous. Further, both balls bowled and logbooks have been shown to be unreliable when quantifying bowling frequency mainly due to adherence issues in reporting [19, 25, 36].

Of the variables of intensity, ball release velocity (BRV) was most reported in the literature and was used as a measure of performance [14, 36, 38, 41–44, 50] or a means of ensuring consistency of effort [31, 40]. As a component of volume, BRV is less meaningful because of the many factors that influence the velocity at which a bowler delivers the ball. Some of these factors include bowler height, the length of various body segments (e.g., arms or legs), physical strength and velocity of the run-up [66–68]. This makes between bowler volume comparisons difficult and the use of BRV less appropriate for bowling programmes targeted at injury prevention.

Aside from a single study that used VAS [12], all subjective ratings of intensity used RPE [13, 16, 23, 35, 38, 47–50, 56]. Although previous research demonstrates that humans can reliably rate their effort and exertion [69], RPE reflects an athletes perceived exertion to an external demand and can be influenced by many factors including previous exercise history [70], personality factors [71], environmental context and nutrition [72]. As a result RPE may be less precise than some objective measures, such as HR or BL [73], assuming that these objective measures are accurately collected using verified methods.

HR and BL were used to quantify physiological response to bowling [39, 47, 48, 56] or fatigue [31, 45, 46] and although it is common for these variables to be used as measures of exercise intensity, it is more accurate to classify them as physiological responses to effort [74, 75]. Further, HR and BL can be impacted by many varied factors, including: exercise training history [76], body mass [77], ambient temperature [78], stress [79], or composition of the playing surface [80]. Therefore, neither are generalisable between bowlers and so are less appropriate as a means of constructing volume-based bowling programmes. Volume, when derived using an appropriate measurement of force (i.e., product of force, frequency and duration), is likely to offer a suitable method to monitor and prescribe training with respect to injury management. However, the current methods used to measure external forces during cricket bowling are limited to laboratory settings [81], which makes it challenging to include force measurements to quantify the demands of training and matches.

Several variables that combine multiple components of frequency, time and intensity to construct a bowling volume were also considered in the literature. The simplest of these involves using the GPS measured variables of distance and velocity [2, 12, 45, 48, 49, 53–59, 64] where volume was calculated as a product of the distance travelled and the velocity involved. Of course, to be useful this requires the assumption that perception of, or physiological responses to, intensity increase as velocity increases, and that this holds true across the population being examined. It is well-established that both HR response and BL can be significantly improved with targeted training [82], but that each individual has a physiological limit to both [83, 84]. Therefore, it is possible that both perception of, and physiological response to, effort may vary between individuals. Once again this leads to a situation where calculation of volumes using GPS measured variables (e.g., distance or velocity), although useful for within bowler comparisons, are insufficient for calculating bowling volumes for the purpose of injury risk management.

Playerload™ is another popular measure of bowling demand that appears in the literature [14, 16, 22, 41, 42, 47, 51, 52, 59] and is used in many different sports [42, 85–89]. PlayerLoad™ uses tri-axial acceleration data to calculate a volume and is purported to provide a measure of how much ‘work’ an athlete does, measured in arbitrary units (AU) [90]. The use of ‘work’ in this way is imprecise, however, as no measure of force [29] is part of the Playerload™ formula [91]. In addition, inconsistencies in calculating Playerload™ and interpreting its meaning have been noted in previous literature [90], demonstrating confusion in its application. Further, previous research has suggested that Playerload™ values have a high level of variability between athletes [92], due to differences in movement patterns for example, making between athlete comparisons unviable. It is unclear how the application of Playerload™ fits with monitoring of bowling volumes other than as a within bowler comparison of volume over time.

The ACWR is used to monitor changes in demand over time using a dual-threshold approach where both too little, and too great, a demand can increase injury risk [93]. However, some conceptual and statistical concerns have been highlighted in a recent study [94] and contradictions exist in the literature as to the usefulness of the ACWR with respect to injury management. For example, Pote and Christie [13] could not identify a relationship between workload and injury risk whereas Warren et al. [18] concluded that large spikes in workload increased injury risk. Inconsistencies in these findings are likely to be partially explained by differences in how ACWR has been calculated in the studies included in this review, where some authors used variables of frequency and time only [18, 27], while others also used RPE or sRPE [13, 15, 23]) when estimating volumes. While it makes sense to customise the variables used to calculate ACWR between sports, such as triathlon and cricket, inconsistencies in variables within a single sport (e.g., cricket) become problematic when used to inform generalised injury management guidelines. While ACWR does provide knowledge of troughs and peaks in bowling volumes, both of which are believed to increase injury risk in fast bowlers [15, 95], the authors of a recent systematic review [95] concluded that despite some studies supporting its use [13, 15, 18], it is yet to be confirmed as useful in managing injury risk. The authors of a recent review propose that the exponentially-weighted moving average model (EWMA) might be more appropriate for determining overall training demands [96]. This model is weighted more heavily towards recent demands, rather than older demands [97], and could be more suitable for fast bowlers than using the rolling average method for determining ACWR as it accounts for the decreasing effects of fitness and fatigue over time.

Commonly used bowling management tools remain important to help understand the number of repetitions performed as well as some aspects of the physiological and psychological demands. In addition, we support the conclusions of other authors [18] who advocate the individualisation of training programmes to provide better outcomes with respect to performance and injury management. There is a need for the inclusion of valid and reliable methods to measure intensity during bowling to quantify training volume and to minimize the risk of injury. We suggest that objective measurements of external force, implementable during training and match play, offer the most promise. One method for deriving forces in this context might be through using inertial measurement units, as described in previous studies on cricket [52, 98]. Callaghan et al. [98] attempted to investigate the use of accelerometers to estimate bowling intensity but suggested that the relationship between segmental accelerometer-derived force curves and GRF experienced during front foot contact was more complex than hypothesised. Therefore, before such a method can be implemented, further validation research is required.

Methods using only variables of frequency or time are not appropriate when measuring demands in bowling and are unlikely to be useful for managing bowling programmes for the purpose of injury management. Further, measures currently used to quantify bowling volume may not be valid to quantify bowling demand because the variables typically used to measure the intensity component have several limitations. Subjective variables and those that measure physiological response to effort are influenced by external factors (e.g., temperature and hydration), and provide little understanding of injury mechanisms. Many factors that can impact on the variables of intensity used in the literature have been identified in this review, which present problems in developing bowling programmes that are uniformly applicable between, or even within, bowlers.

While elite cohorts have been most often studied, the potential exists to develop monitoring tools that can be used with non-elite bowlers. Currently, it is less likely that sub-elite and grassroot cohorts have access to the technical or other resources necessary to consistently and effectively measure and monitor bowling demands. Where these cohorts do monitor bowling demands they are limited to using the simple and mainly subjective methods we have identified in this review. There is an opportunity for future research to explore methods for measuring bowling demands that can benefit cricketers of all ages and abilities.

### Limitations

Although 48 studies were included in this systematic review, only thirteen linked FITT-VP variables to injury incidence. Further, the variables used in these studies have several limitations including impacts from environmental factors such as temperature and ground hardness, or hydration levels of participants. Thus, drawing conclusions from these studies is difficult and more research is needed linking bowling volumes to injury. Lastly, although the literature search strategy found significantly more articles than other recent similar systematic reviews in cricket [4, 99, 100] it is acknowledged that it is possible some relevant studies were missed (for example, those not available in English language or those in the grey literature). However, we feel confident that most relevant studies have been identified, and in the inferences drawn from the included studies.

## Conclusion

Current methods for measuring demand in bowling are typically imprecise or insufficient in providing a foundation to build bowling volume programmes on. Critically, measures of intensity that have been used to calculate bowling volumes are either not representative of real forces or are not able to be generalised to a bowling population. This does not facilitate the writing of bowling guidelines to reduce injury risk. Considering this, more appropriate intensity measures that incorporate a measure of force would be desirable in monitoring bowling volumes. There is an opportunity for further research to develop more precise measures of bowling intensity and volume that may lead to sustainable reductions in injury risk, incidence, and prevalence.

## Data Availability

All data generated or analysed during this study are included in this published article.
